# Acquired Traits Contribute More to Drought Tolerance in Wheat Than in Rice

**DOI:** 10.34133/2020/5905371

**Published:** 2020-06-12

**Authors:** Preethi Vijayaraghavareddy, Ramu S. Vemanna, Xinyou Yin, Paul C. Struik, Udayakumar Makarla, Sheshshayee Sreeman

**Affiliations:** ^1^Department of Crop Physiology, University of Agricultural Sciences, Bengaluru, India; ^2^Centre for Crop Systems Analysis, Department of Plant Sciences, Wageningen University & Research, PO Box 430, 6700 AK Wageningen, Netherlands; ^3^Regional Centre for Biotechnology, Faridabad, Haryana, India

## Abstract

Drought tolerance is governed by constitutive and acquired traits. Combining them has relevance for sustaining crop productivity under drought. Mild levels of stress induce specific mechanisms that protect metabolism when stress becomes severe. Here, we report a comparative assessment of “acquired drought tolerance (ADT)” traits in two rice cultivars, IR64 (drought susceptible) and Apo (tolerant), and a drought-tolerant wheat cultivar, Weebill. Young seedlings were exposed to progressive concentrations of methyl viologen (MV), a stress inducer, before transferring to a severe concentration. “Induced” seedlings showed higher tolerance and recovery growth than seedlings exposed directly to severe stress. A novel phenomic platform with an automated irrigation system was used for precisely imposing soil moisture stress to capture ADT traits during the vegetative stage. Gradual progression of drought was achieved through a software-controlled automated irrigation facility. This facility allowed the maintenance of the same level of soil moisture irrespective of differences in transpiration, and hence, this platform provided the most appropriate method to assess ADT traits. Total biomass decreased more in IR64 than in Apo. The wheat cultivar showed lower levels of damage and higher recovery growth even compared to Apo. Expression of ROS-scavenging enzymes and drought-responsive genes was significantly higher in Apo than in IR64, but differences were only marginal between Apo and Weebill. The wheat cultivar showed significantly higher stomatal conductance, carbon gain, and biomass than the rice cultivars, under drought. These differences in ADT traits between cultivars as well as between species can be utilised for improving drought tolerance in crop plants.

## 1. Introduction

Rice (*Oryza sativa* L.), being a semiaquatic species, is generally cultivated under puddle conditions. However, with the impending climate change combined with domestic and industrial demands for fresh water, cultivation of rice in the conventional puddle system is fast becoming less feasible [1]. This necessitates development of suitable water-saving agronomic practices to save water. Practices such as semi-irrigated aerobic cultivation are known to save more than half of the water used and avoid the destruction of soil structure while puddling [2]. However, a concomitant yield loss by more than 40% when grown under the aerobic system has been observed [3, 4]. Therefore, we should attempt to enhance the genetic potential that can produce more biomass and yield with reduced water requirement.

Selection for higher absolute yields under water-limiting conditions was one of the major approaches for improving rice productivity. Although this approach provided initial dividends, further improvement in rice productivity has become hard to achieve [4]. To overcome this bottleneck, a focused trait-based breeding approach has been proposed and is being widely adapted [5]. This led to a significant increase in enumerating physiological and morphological traits and establishing their functional relevance in conferring stress tolerance in rice plants [4]. Depending on the level of organization and response to external stimuli, drought-adaptive traits are broadly classified as “constitutive,” “responsive,” and “acquired” tolerance traits [4]. Traits that are always expressed such as root number, stoma number, specific leaf area, and epicuticular waxes are referred to as "constitutive traits or integral traits" [6]. Traits that are expressed always but would significantly change in response to stress such as root length and osmotic adjustment are often referred to as “responsive” traits [4]. On the other hand, “acquired tolerance traits” are conspicuously absent under well-watered conditions but get upregulated when plants experience gradual induction of a stress [7]. *De novo* gene expression has been shown to increase when plants experience mild stress levels. This upregulation of gene expression is known to induce specific mechanisms that provide protection to cellular metabolism when stress gets severe. Acquired tolerance is therefore associated with the upregulation of several diverse processes such as maintenance of redox homeostasis, regulation of gene expression, protein turnover, DNA/protein repair mechanisms, osmotic adjustment, and membrane stability including specific metabolomic changes [8]. Maintenance of cellular metabolism is dependent upon the abilities of the plant to sustain positive tissue turgor and to maintain cell membrane integrity. While turgor maintenance is governed mostly by constitutive traits such as root and leaf surface characters [9], maintenance of cellular metabolism even under decreasing tissue turgor is more associated with acquired tolerance traits [9].

Maintenance of cell membrane integrity is in turn dependent on the ability to manage oxidative stress [8]. Increased production of reactive oxygen species (ROS) is an inevitable consequence of stress encountered by plants. Naturally, plants have evolved several mechanisms to balance the ROS homeostasis under stress through upscaling several ROS-scavenging mechanisms [4]. Increased activity of some key ROS-scavenging enzymes like ascorbate peroxidase (APX), superoxide dismutase (SOD), glutathione peroxidase (GPX), dehydroascorbate reductase (DHAR), and catalase (CAT) has been reported in many species. Wheat genotypes that show increased activity of these enzymes also display considerable stress tolerance [10]. Interestingly, these protective mechanisms are generally upregulated when plants experience mild levels of stress and hence develop tolerance to stress when the severity increases. These mechanisms that provide acquired tolerance display large genetic diversity, which also depends on the type of stress and the progression of stress occurrence [11, 12].

The premise of this investigation is based on the hypothesis that a combination of constitutive traits with acquired tolerance traits would comprehensively improve drought adaptation. Methods for determining constitutive traits have been developed under both laboratory and field conditions [4, 6]. Methods for determining acquired tolerance require the induction of young seedlings with a mild level of stress before transferring the seedlings to severe or lethal stress levels. A few laboratory experimental protocols have been developed where, young seedlings are induced with a gradually increasing temperature until it reaches lethal levels. This method, referred to as Temperature Induction Response (TIR), is a convenient assay for assessing acquired tolerance [13, 14]. Similarly, inducing changes in cellular metabolism with the use of specific stress “inducers” such as methyl viologen (MV) is also a simple approach to assess acquired tolerance at the seedling stage [15]. Many studies have documented significant genetic variability in acquired tolerance levels using these experimental protocols. Furthermore, several studies reported that crop genotypes with higher acquired tolerance recorded improved growth and performance under drought stress conditions. Rice genotypes with higher acquired tolerance had higher spikelet fertility under stress and hence were associated with a superior yield [9].

Realising the importance of acquired tolerance in drought adaptation, several methods to quantify this trait were developed [16, 17]. Although these methods provide clues on genetic variations in the propensity to respond to stress, these methods rely on the induction of response through priming young seedlings with temperature and/or stress-inducing molecules. Examining the stress response by providing drought itself as an inducer has been the most important limitation.

To capture these stress-responsive mechanisms, precise imposition and accurate maintenance of a specific stress regime are of paramount importance. Advancements in high-throughput phenotyping approaches led to the establishment of high-end phenomic platforms that are increasingly being used for imposing stress and for capturing genetic variability in stress responses [18]. Gravimetric principles have, by and large, been the approach for determining water lost by a potted plant. Automated water dispensing systems have also been developed in these high-end phenomic platforms to maintain a specific soil moisture status. However, these platforms are limited by the number of gravimetric determination of water loss in a day. Thus, mimicking the progression of stress in natural conditions still remains a major challenge. We developed a novel phenomic platform with an automated irrigation system that is interfaced with transpirational water loss. Thus, the system maintains the water content in the soil within 1% of the fixed field capacity and hence represents an excellent system to examine stress response of plants. The most important feature of this facility is its ability to progressively decrease the water added so as to bring the soil to a specified level of soil moisture stress. This transpiration-interfaced automated irrigation system can therefore accurately mimic a field-like drought progression scenario. The other most prominent feature of this facility is its ability to maintain the same level of stress despite any possible differences in transpiration rates and hence represents an excellent system to compare stress responses of genotypes within as well as across species. Therefore, this system provides an opportunity for a comparative assessment of rice and wheat *(Triticum aestivum L.)* for the differences in their acquired tolerance levels.

Rice and wheat are the two most extensively consumed C_3_ cereals, with phenomenal differences in water use patterns that render wheat to be better adapted to drought than rice. Therefore, growing rice like wheat would have phenomenal significance in saving water. Comparing the stress response of these two cereals to water limitation would provide scientific insights to prepare rice for limited water resources. Rice and wheat are known to differ in several morphophysiological traits and hence have reduced water requirement. While large differences in water absorption through root system architecture, metabolic status, etc., are well known to be superior in wheat [19–21], no systematic study has ever been done to assess the differences in acquired tolerance between these cereal species. We hypothesise that when acquired tolerance traits are combined with constitutive traits such as roots and water use efficiency, such genotypes would have comprehensively higher adaptability to drought. We examined rice and wheat cultivars for the differences in acquired tolerance besides other traits. The major intent of this study was to examine the relevance of acquired tolerance in imparting drought adaptation, by comparing tolerant and susceptible rice cultivars and also rice and wheat. An initial experiment was conducted to assess the best suitable stress imposition approach (induction and lethal) to study acquired mechanisms of rice and wheat using MV which is a potent inducer of oxidative stress. Further experiments were done using the phenomic facility by following an induction protocol. The results clearly demonstrate the superior drought tolerance in wheat compared to even a known drought-adaptive cultivar of rice.

## 2. Materials and Methods

### 2.1. Plant Material and Approach

Two rice cultivars, IR64 and Apo, and one wheat cultivar, Weebill, were used in the experiments. IR64 is a drought-susceptible, high-yielding, lowland rice cultivar, and Apo is a drought-tolerant, high-yielding, and aerobic rice cultivar. The wheat cultivar Weebill is known to be drought tolerant [21]. Two types of a stress imposition method and three independent experiments were conducted at the research facilities of the University of Agricultural Sciences, Bengaluru, India (12°58′N, 77°35′E): the first one used a chemical stress inducer with young seedlings (48 h old), and the second was to examine the drought stress response both at the seedling (15 days after sowing (DAS)) and vegetative (35 DAS) stages. Drought treatment was imposed using the phenomic platform with an automatic irrigation capability (described later).

### 2.2. Experiment 1: Stress Imposition Using Methyl Viologen

Methyl viologen (MV) is an artificial electron donor to both mitochondrial and chloroplast electron transport [22–24] and hence generates reactive oxygen species (ROS). At high concentrations, MV can be lethal. To assess the effect of MV at the young seedling stage, 48 h-old uniformly germinated seedlings were used. An induction protocol was developed by treating young seedlings with progressively increasing concentrations of MV before transferring the seedlings to a “severe” concentration. To determine this lethal concentration, uniformly germinated rice seeds of cv. IR64 were spread on 13 cm diameter Petri plates with two layers of wet filter papers. Each plate had 10 seedlings. Plates with 48 h-old seedlings were exposed to different concentrations of MV to determine the “severe stress” concentration of MV, i.e., the concentration at which about 95% of the seedlings died (Fig. S1). A concentration of 10 *μ*M MV was found to be “severe” or lethal. An induction protocol was developed that involved transferring the 48 h-old seedlings to sequentially increasing concentrations of MV, i.e., 2, 4, 6, and 8 *μ*M of MV. Seedlings were allowed to stay for a duration of 3 hours in each of the concentrations before being transferred to the next higher concentration, and subsequently, the “induced” seedlings were transferred to 10 *μ*M concentration of MV (lethal stress). Three plates were maintained for each treatment, and a completely randomized design was adopted for statistical analysis. To every plate, 10 ml of each concentration of MV was added. One set of seedlings was directly transferred to 10 *μ*M MV, to represent the “severe stress” treatment, while a separate set of seedlings was continuously kept in plates wetted with distilled water to represent the “absolute control.” Seedlings in all treatments were exposed to 600 *μ*mol m^−2^ s^−1^ of light intensity in a controlled growth chamber with an air temperature maintained at 30°C with 60% RH throughout the experiment period. Shoot and root lengths were recorded between 10:00 and 12:00 hours at the end of the stress period (72 hours from the stress imposition). Recovery growth was assessed 48 hours after transferring the seedlings from the induction and severe stress treatments to distilled water. Comparison was made between induction and lethal treatments within cultivars. Measurements of membrane damage, reactive oxygen species (ROS), and reactive carbonyl compound (RCC) production, antioxidant activities, etc., were made with the seedlings subjected to induction stress, and the results were compared in relation to that of the seedlings which were directly exposed to sever stress.

#### 2.2.1. Quantification of Superoxide (O_2_^−^) by Nitrotetrazolium Blue Chloride (NBT) Staining

Seedlings were transferred to NBT solution to detect superoxide radicals. Seedlings were immersed in 0.2% NBT solution dissolved in 50 mM sodium phosphate buffer with a pH of 7.5. NBT reacts with O_2_^−^ to form a dark-blue insoluble formazan compound. The seedlings were transferred to a bleaching solution to remove chlorophyll. Tissues were ground in 0.1% acetic acid [16]. The samples were centrifuged at 10,000 rpm for 10 min, and absorbance was read at 560 nm.

#### 2.2.2. Quantification of Hydroxyl (^∙^OH) Radicals

The presence of hydroxyl radicals in seedlings was quantified using the method described by [25]. Seedlings exposed to MV stress as described above were immediately transferred and homogenized in 1.2 ml of 50 mM sodium phosphate buffer (pH 7.0) and centrifuged at 12,000 rpm at 4°C for 10 min. The supernatant was collected (0.5 ml) and 0.5 ml of 50 mM of sodium phosphate buffer (pH 7.0) and 1 ml of 25 mM sodium phosphate buffer containing 2.5 mM 2-deoxyribose were added to the supernatant, and this mixture was incubated at 35°C in the dark for 1 h. After incubation, 1 ml of 1% thiobarbituric acid (TBA, Sigma, USA) and 1 ml of glacial acetic acid were added and the mixture was boiled for 10 min and cooled immediately on an ice bath. Absorbance was recorded at 532 nm.

#### 2.2.3. Measurement of Malondialdehyde (MDA) Content

Fresh leaf tissue (0.2 g) was homogenized in 0.1% trichloroacetic acid (TCA) and centrifuged at 14,000 rpm for 15 min. 2.5 ml of 0.5% thiobarbituric acid (TBA) in 20% TCA was added to 1.0 ml of the supernatant, and the mixture was incubated at 95°C in a water bath. After 30 min of incubation, it was cooled immediately and centrifuged at 10,000 rpm for 30 min. Absorbance was determined at 532 and 600 nm, and MDA concentration was estimated by subtracting the OD at 600 nm from the OD at 532 nm as a correction for nonspecific turbidity [26].

#### 2.2.4. Quantification of Methylglyoxal (MG)

Fresh tissue (100 mg) was collected from both control and treated seedlings and ground in a known volume of distilled water. The extract was centrifuged at 11,000 rpm at 4°C for 10 min. 250 *μ*l of 7.2 mM 1,2-diaminobenzene and 100 *μ*l of 5 M perchloric acid were added to the supernatant (650 *μ*l). The absorbance in the mixture was measured at 336 nm using a spectrophotometer (SpectraMax Plus 384, Spinco Biotech Pvt Ltd, Bangalore) [26].

#### 2.2.5. *α*,*α*-Diphenyl-*β*-picryl-hydrazyl (DPPH) Assay for Total Scavenging Activity

The total free radical scavenging activity was measured as described by McCune and Johns [27]. The reaction mixture containing 1 ml of the methanolic leaf extract and 1 ml of DPPH solution (0.3 mM) was incubated in the dark for 10 min. Absorbance was read at 517 nm, and percent inhibition was calculated over the control.

#### 2.2.6. Quantification of Superoxide Dismutase (SOD) Activity

SOD activity was quantified by using the photochemical NBT method as described by Beyer et al. [28]. In this method, assay buffer containing L-methionine (300 mg/10 ml), NBT-2HCl (14.1 mg/10 ml), and Triton X-100 (1%) were added to a glass tube. 20 *μ*l of the sample extracted using phosphate buffer was delivered to this mixture. To initiate the reaction, 10 *μ*l of riboflavin (4.4 mg in 100 ml) was added. The tube was illuminated at a light intensity of 600 *μ*mol m^−2^ s^−1^ along with the control (without the sample). Absorbance at 560 nm was recorded in all the tubes, and a percentage decrease in NBT reduction due to SOD activity was calculated. The percent increase in SOD activity due to the stress effect was calculated over the control.

#### 2.2.7. Quantification of Membrane Damage by the Evans Blue Technique

Seedlings exposed to MV stress were immediately transferred to 5 ml tubes containing 0.25 g of Evans blue dye prepared in 0.1 M CaCl_2_ (pH 5.6). Seedlings were immersed completely in Evans blue dye and incubated for one hour in the dark. After incubation, seedlings were washed thoroughly using distilled water to remove any stain adhered to the surface. Later, seedlings were transferred to 2 ml Eppendorf containing 1 ml of 1% sodium dodecyl sulphate (SDS) and ground using a tissue lyser. Suspension was centrifuged at 10,000 rpm for 10 min. The supernatant was collected, and absorbance was recorded at 600 nm [17].

#### 2.2.8. Quantification of Proline Content

Fresh tissue (0.1 g) was homogenized in 3% sulfosalicylic acid. The homogenate was centrifuged at 6000 g for 20 min. Acids ninhydrin (2 ml) (1.25 g of ninhydrin, 20 ml of 6 M orthophosphoric acid, and 30 ml glacial acetic acid) and glacial acetic acid (2 ml) were added to a 2 ml aliquot and the mixture was boiled at 100°C for 1 h, and the reaction was terminated in an ice bath. 4 ml of toluene was added to this mixture to extract chromophores from the aqueous phase, and absorbance was read at 520 nm. The proline content was determined from a standard curve and expressed as *μ*mol g^−1^ fresh weight [29].

### 2.3. Experiment 2: Drought Stress Imposition Using an Automated Drought Simulator Phenotyping Platform

Two independent experiments were conducted at the minilysimeter- (MLM-) based drought simulator platform to examine the response of plants to drought stress and to assess acquired tolerance. The first experiment (experiment 2A) was carried out using 15-day-old seedlings in containers (10 l capacity) filled with 10 kg of a rooting mixture comprising red sandy-loam soil mixed with farm yard manure in a 3 : 1 (*W*/*W*) proportion. The second experiment (experiment 2B) was carried out using 35-day-old plants using larger containers (20 l capacity) filled with 20 kg of a rooting mixture.

**Figure 1:**
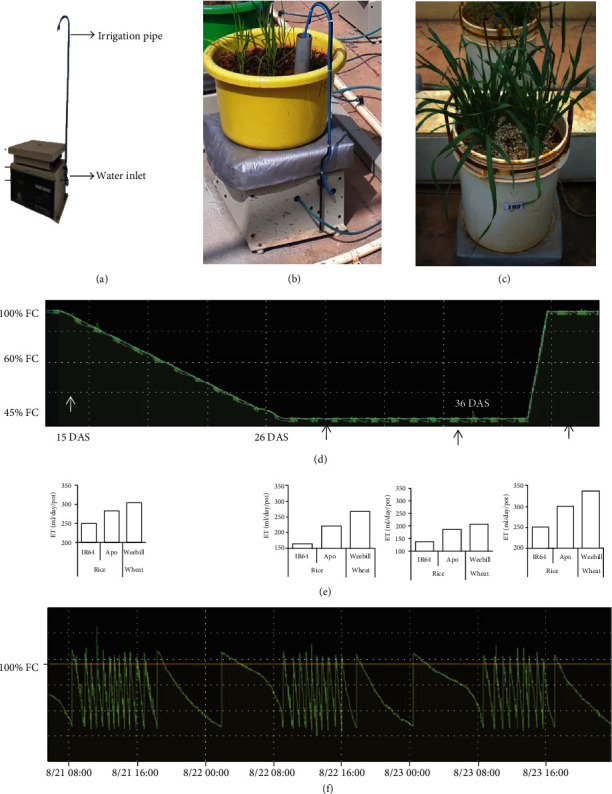
Drought stress imposition protocol for rice and wheat using a plant phenomic platform. (a) Minilysimeter with an irrigation pipe. (b) Pots of 10 l capacity and (c) 20 l capacity were kept on lysimeters with the automated irrigation facility. (d) Gradual moisture stress (dry-down) was imposed with a reduction of 5% of FC per day to reach 45% FC which was then elevated to 100% FC after 10 days. (e) Bar graph indicating evapotranspiration data of IR64, Apo, and the wheat cultivar Weebill for one day. (f) The graph is indicating real-time water transpired and added per day.

Acquired tolerance for drought stress can be measured only when the plants are exposed to a gradually progressing stress. The novel phenomic platform established at our centre was utilised to generate a specific level of drought stress in container-grown plants. This facility consists of containers (minilysimeters (MLM)) placed on individual load cell balances and is capable of determining the weights in real time. The load cell balances are capable of determining weights up to 50 kg with an accuracy of ±50 g (Figures 1(a)–1(c)). The weight of containers decreases due to evapotranspiration, and hence, this gravimetric approach represents a very accurate method to determine water used by plants. Custom-designed software recognises this weight reduction and commands a solenoid value to dispense accurate volumes of water to replenish the water lost by evapotranspiration. The irrigation water is dispensed into a 2 cm diameter tube that has a round coil of tube at the middle of the container. The tubes are perforated to facilitate uniform distribution of water in the container. This setup ensures the maintenance of soil moisture within 1% of the set water regime in the container. The most important feature of this software is its capacity to bring down the water level at a rate determined by the user. This transpiration-interfaced irrigation software dispenses a predesignated proportion of water so as to provide an accurate rate of depletion of water. This “dry-down” protocol is capable of precisely mimicking the field conditions as well as creating a gradually developing stress condition (Figure 1(d)). The software controlling the automated irrigation is also capable of maintaining the soil moisture status at any notified level, and hence, this system is the most appropriate method for assessing acquired tolerance (patent application number is 201941028751). The other major feature of this automated irrigation facility is its ability to precisely maintain a highly comparable tissue water status for cultivars independent of any differences in their transpiration rates (Figures 1(e) and 1(f)). The entire facility is established under naturally varying conditions. The weather parameters that prevailed during the experimental period were obtained from the weather station of the university, located around 300 m from the experimental plot (Tables S1 and S2).

#### 2.3.1. Experiment 2A: Stress Imposition at the Seedling Stage

Stress imposition was initiated at 15 days after sowing (DAS) for rice and wheat seedlings by automated irrigation. A set of five containers were maintained at 100% field capacity (FC) while another five containers were subjected to gradual stress. The software was tuned to ensure a 5% reduction in soil water status per day irrespective of the differences in evapotranspiration. The containers designated as “water stressed” reached 45% FC on the 11^th^ day after stress imposition. Stress level was maintained at 45% FC for an additional 10 days. Afterwards, soil water status was elevated to 100% FC for recovery. To measure relative water content (RWC), leaf discs were made from both control and stress samples and fresh weight was recorded. Leaf discs were floated on distilled water in Petri plates for 5 h, and turgid weight was recorded. Leaf discs were oven dried at 65°C for three days, and dry weight was recorded. The RWC was calculated using the formula: RWC% = ((Freshweight − dryweight)/(turgidweight − dryweight)) × 100. Total chlorophyll content was measured using the acetone : DMSO (1 : 1) method. Leaf discs were incubated in 10 ml of acetone : DMSO (1 : 1) overnight in the dark. Absorbance was measured at 663, 645, and 652 nm to calculate total chlorophyll content as described by Arnon [30]. MDA and total proline were measured in replicates of three using the protocol described above in the second leaf collected during bright sunshine hours (10:00 to 11:00 hours). Plant height, tiller number, and biomass were measured a week after recovery in three replicates.

To prove that gradual drought stress upregulates drought-responsive scavenging enzymes, ascorbate peroxidase (APX), FeSOD, and catalase (CAT), aldo-keto reductase (AKR1) *Δ*1-pyrroline-5-carboxylate synthase (P5CS) expression levels were measured. Along with scavengers, ER stress-responsive genes, binding protein (BiP1) and calreticulin (CRT1) activity were also measured by expression studies. Leaf tissues from seedlings grown at 100% and 45% FC in replicates of three were used to extract total RNA using the phenol-chloroform method according to Datta et al. [31], and cDNA was synthesized by oligo (dT) primers using Moloney murine leukaemia virus reverse transcriptase (MMLV-RT; MBI Fermentas, Hanover, MD). The cDNA pool was used as a template to perform RT-PCR analysis according to the manufacturer's protocol (Opticon 2; MJ Research, USA, and MJ Bioworks Inc.). The quantitative real-time RT-PCR was performed with the fluorescent dye SYBR Green (TAKARA SYBR Green qPCR Kit). The relative expression levels of the selected genes under a given stress condition were calculated using the comparative threshold method. The PCR was performed with SYBR dye, and the PCR conditions were 94°C for 3 min, 25 cycles of 94°C for 30 s, 52–58°C for 30 s, and 72°C for 40 s, and a final extension of 72°C for 5 min. Ubiquitin was used as an internal control for normalization.

#### 2.3.2. Experiment 2B: Stress Imposition at the Vegetative Stage

In a set of 10 containers, the plant population was progressively thinned to maintain two uniform and healthy plants per container by 25 DAS. Stress imposition was initiated on the 35^th^ DAS, as described above. A set of five containers for each cultivar was designated as “45% FC” while the other five were maintained at 100% FC (well-watered). The automated irrigation system of the drought simulator platform regulates the water added to containers, and plants reached 45% FC on the 11^th^ day after stress imposition. Soil water status was maintained at 45% FC for additional 10 days and later elevated to 100% FC and maintained until maturity. During the stress period, photosynthetic rate and stomatal conductance were measured using a portable photosynthetic system (LICOR 6400, Lincoln, Nebraska, USA). RWC and MDA content were measured in the second leaf collected during bright sunshine hours (10:00 to 11:00 hours) using the protocol described above. The canopy temperature was measured in 100% and 45% FC plants using the Fluke thermal imaging system. Images taken from the canopy using a Fluke camera were analysed using SmartView software. Data was exported to Microsoft Excel to analyse all the data points of the image. To avoid background noise, the canopy was outlined to arrive at mean temperature [32]. At harvest, from three replicates, shoot, leaves, and roots were separated, oven dried at 65°C for three days, and weighed; values were computed to arrive at total leaf area and total biomass.

### 2.4. Statistical Analysis

All the data were analysed to check statistical significance using Genstat (15^th^ edition) (http://www.genstat.co.uk/). The goal of this investigation was to compare the effect of “induction” stress on plant performance when stress levels become severe. For this comparison, a one-way statistical algorithm was used in experiment 1, where various parameters of induced and severely stressed plants were compared. Further, the comparisons were also made between the two rice cultivars (Apo and IR64) and between the tolerant rice cultivar Apo and wheat cultivar, Weebill in induction treatment only. In experiment 1, methyl viologen, a stress inducer, was used. In experiment 2, the comparison was made at 45%FC between the two cultivars of rice and between the tolerant rice cultivar Apo and wheat cultivar, Weebill. Plants were exposed to progressive drought stress using the automated phenomic platform, and the statistical significance of several parameters was compared by performing a one-way ANOVA. Comparisons were also made between Apo and Weebill using the same algorithm.

## 3. Results

### 3.1. Wheat Has Higher Oxidative Stress Tolerance Than Rice

**Figure 2:**
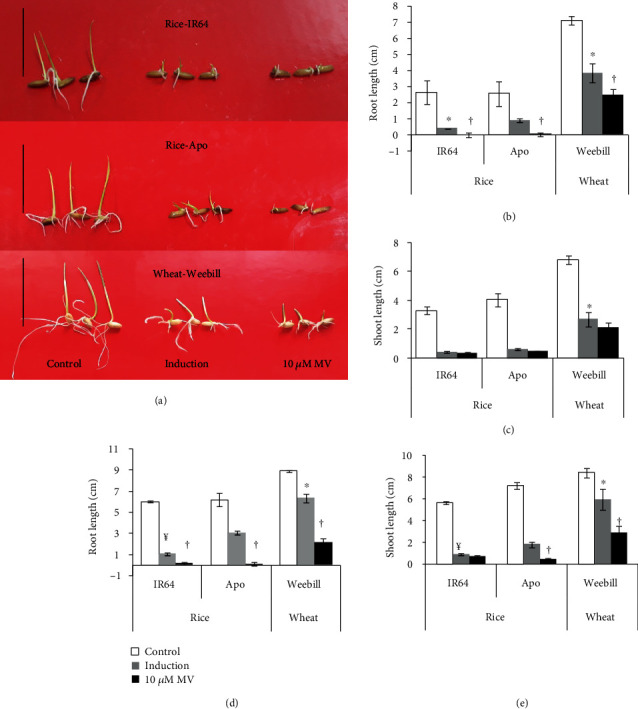
Effect of methyl viologen (MV) on plant growth in induction and severe stress treatments (10 *μ*M) in both rice (IR64 and Apo) cultivars and the wheat (Weebill) cultivar (Experiment 1). Effect of MV on (a) plant growth, (b) root length, and (c) shoot length measured at the end of the stress period (72 hours after stress imposition). Seedlings from all the treatments were subjected to recovery growth for two days, and (d) root length and (e) shoot length were measured after the recovery period. Scale bar = 8cm. ^†^Significant difference between induction and severe stress treatments within cultivars at *p* ≤ 0.05. ^¥^Significant difference between rice cultivars (IR64 and Apo) in the induction treatment at *p* ≤ 0.05. ^∗^Significant difference between tolerant rice cv. Apo and wheat cv. Weebill in the induction treatment at *p* ≤ 0.05.

A significant reduction in growth was observed in MV-treated seedlings of the rice cultivars, with a strong reduction in root length, especially in the susceptible cv. IR64 at the end of the stress period (Figures 2(a) and 2(b) and Table S3a). The wheat cultivar, Weebill, on the other hand showed better tolerance with sustained shoot and root growth compared to the tolerant rice cv. Apo (Figures 2(b) and 2(c)). Neither of the two rice cultivars survived the severe stress treatment, but wheat proved to be more tolerant to the severe stress treatment (Figure 2(a)). In the induction treatment, recovery growth of seedlings was significantly superior to that of seedlings directly exposed to the severe stress treatment for both cereals (Figures 2(d) and 2(e)). Rice cv. Apo had a higher recovery when stress was gradually induced than cv. IR64. Wheat had significantly more root and shoot growth than the tolerant rice cultivar Apo when the stress was gradually induced (Figures 2(d) and 2(e) and Table S3b).

Generation of reactive oxygen species (ROS) is an inevitable consequence of the stress response, and the ability to scavenge these ROS is considered a stress-adaptive mechanism. There was a significant increase in O_2_^−^ in all the cultivars of both rice and wheat (Table 1). However, the increase in ROS production was significantly lower in the seedlings receiving induction treatment than in the seedlings directly exposed to severe stress treatment. Similar observations were made for ^∙^OH accumulation. However, between the two rice cultivars, there was a marginally lower accumulation of O_2_^−^ and ^∙^OH in cv. Apo than in cv. IR64 in the induction treatment. Weebill recorded the lowest levels of O_2_^−^, while ^∙^OH accumulation was not significantly different in the induction treatment than that in tolerant rice cv. Apo (Table 1).

Lipid peroxidation and glycation lead to the production of several cytotoxic compounds called reactive carbonyl compounds (RCCs), which are consequences of oxidative stress. Seedlings exposed directly to svere levels of MV experience an oxidative burst leading to a significant increase in MDA and MG content in the susceptible rice cv. IR64 compared to Apo (Table 1). But when seedlings were induced with mild stress levels of MV, the increase in MDA and MG were significantly lower and it was lowest in Weebill. The MDA and MG levels in the wheat cultivar were lower than those in the tolerant rice cultivar, Apo. These data indicate the enhancement of acclimation response upon induction (Table 1). Lipid peroxidation and glycation processes are known to damage the cell membrane stability. Evans blue staining, a sensitive assay to determine membrane damage, revealed that Apo experienced less membrane damage compared with IR64 indicating a superior induction response in the tolerant cultivar. The membrane damage of the wheat cultivar Weebill was lower than that of Apo indicating an even better protective mechanism operating in wheat compared to rice (Table 1).

**Figure 3:**
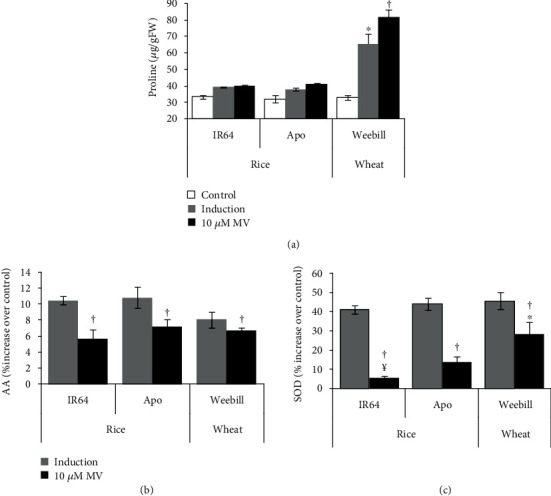
Effect of methyl viologen (MV) on scavenging activity in the induction treatment compared to the severe stress treatment (10 *μ*M MV) in both rice (IR64 and Apo) cultivars and the wheat (Weebill) cultivar (Experiment 1). (a) Proline content. (b) Percent increase in antioxidant activity (AA) over the control measured by the DPPH method. (c) Percent increase in superoxide dismutase (SOD) activity over the control measured at the end of the stress period. ^†^Significant difference between induction and severe stress treatments within cultivars at *p* ≤ 0.05. ^¥^Significant difference between rice cultivars (IR64 and Apo) in the induction treatment at *p* ≤ 0.05. ^∗^Significant difference between tolerant rice cv. Apo and wheat cv. Weebill in the induction treatment at *p* ≤ 0.05.

As a stress response, ROS-scavenging antioxidants and enzymes increased in both induction and severe stress treatments as compared to the control for both cereals. Proline content was not significantly different between the induction and severe stress treatments in rice (Figure 3(a)). But fold change in proline was significantly higher for wheat in severe stress compared to induction treatment (Figure 3(a)). To assess the acquired tolerance through their abilities to scavenge ROS, a percent increase in total antioxidant activity and SOD activity was measured (Figures 3(b) and 3(c)). Both rice cultivars showed significant increases in total antioxidant and SOD activities when induced with gradually increasing stress levels. The levels of induction of SOD activity and total antioxidants in wheat were comparable with those in the tolerant rice cultivar Apo (Figure 3(c)). But the SOD activity in the wheat cultivar was significantly higher even in the severe stress treatment which indicates a greater ability to scavenge ROS in wheat compared to rice (Figure 3(c)).

### 3.2. Variability in Acquired Mechanisms at the Seedling Stage

**Figure 4:**
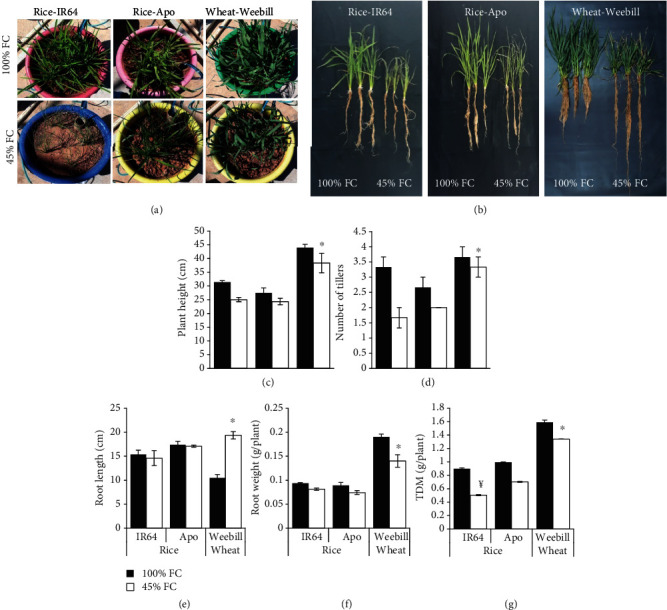
Effect of gradual induction of moisture stress on morphophysiological parameters of IR64 and Apo (rice) and Weebill (wheat) (15 DAS—experiment 2A). (a) Phenotype of plants in 100% FC and 45% FC at the end of the stress period and (b) 7 days after recovery. Effects of moisture stress on (c) plant height, (d) number of tillers, (e) root length, (f) root weight, and (g) total dry matter (TDM) measured after recovery. Scale bar = 45cm. ^¥^Significant difference between rice cultivars (IR64 and Apo) in the induction treatment at *p* ≤ 0.05. ^∗^Significant difference between tolerant rice cv. Apo and wheat cv. Weebill in the induction treatment at *p* ≤ 0.05.

Rice and wheat seedlings (15 days old) were subjected to gradual drought stress of 45% FC using the phenotyping platform (Figure 4(a)). The gradual stress imposition protocol that was adopted in these experiments ensured that the seedlings were “induced” with milder stress levels before reaching the target stress of 45% FC. At the end of the stress period, cv. IR64 was more wilted indicating poor tolerance to stress (Figure 4(a)). Morphophysiological parameters were measured a week after recovery (Figure 4(b)). Although both the rice cultivars had a similar reduction in plant height at 45% FC, the reduction in the tiller number was marginally higher for cv. IR64 than for cv. Apo. But wheat was unaffected by a reduction in soil moisture in terms of both plant height and tiller number (Figures 4(c) and 4(d)). No significant reduction in root length and weight was recorded for rice cultivars. Interestingly, in wheat, although root biomass decreased under stress, a significant increase in root length was observed (Figures 4(e) and 4(f)). Wheat plants recovered faster with higher biomass compared to cv. Apo (Figure 4(g)). The total biomass recorded after recovery was significantly less in rice cv. IR64 than in rice cv. Apo, while wheat recorded significantly higher total biomass accompanied with the least percent reduction in total biomass even when compared with the tolerant rice cv. Apo at 45% FC (Figure 4(g)).

The automated irrigation system precisely maintains a specific soil water status by gravimetrically recognising the water loss from each individual container. Therefore, maintenance of a given moisture status irrespective of differences in water use or root traits is the most prominent feature of the stress imposition system adopted. Accordingly, the relative water content (RWC) did not differ significantly at 45% FC between species (Table 2). Interestingly, no reduction was found in the chlorophyll content relative to 100% FC in both rice and wheat. However, the wheat cultivar Weebill recorded higher chlorophyll content than the rice cultivars in both well-watered and 45% FC conditions (Table 2). As a biomarker that reflects stress, the MDA content increased significantly in both rice cultivars, but more so in IR64 than in Apo. Wheat had much lower MDA levels than the drought-tolerant rice cultivar Apo (Table 2). The highest increase in the proline content was seen in wheat and was marginally higher even under well-watered conditions compared to that in rice. Between rice cultivars, IR64 had a higher increase in proline than tolerant rice cv. Apo in 45% FC (Table 2).

### 3.3. Upregulation of Drought-Responsive Genes Was Crucial for Acquired Mechanisms

**Figure 5:**
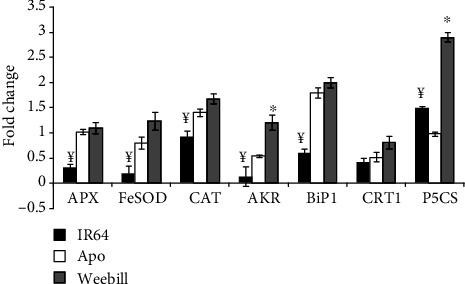
Real-time quantitative RT-PCR analysis for drought-responsive genes in cv. IR64 and cv. Apo (rice cultivars) and cv. Weebill (wheat). The second leaf of 100% FC and 45% FC treatments was used from experiment 2A. ^¥^Significant difference between rice cultivars (IR64 and Apo) in the induction treatment at *p* ≤ 0.05. ^∗^Significant difference between tolerant rice cv. Apo and wheat cv. Weebill in the induction treatment at *p* ≤ 0.05.

The tolerant rice cv. Apo showed significant increases in drought-responsive genes. The ROS-scavenging enzymes, ascorbate peroxidase (APX), FeSOD, and catalase (CAT), are known to balance the ROS in the system by detoxifying and were significantly upregulated in cv. Apo compared to cv. IR64 under stress. Further, reactive carbonyl compound scavengers, aldo-keto reductases (AKR1), showed significant upregulation of transcript in cv. Apo than in cv. IR64 (Figure 5). An endoplasmic reticulum (ER) chaperon-binding protein (BiP1) activity was more enhanced in cv. Apo than in cv. IR64. But another ER stress-responsive gene calreticulin (CRT1) activity did not show any variation between IR64 and Apo (Figure 5). However, interestingly, the gene involved in proline biosynthesis *Δ*1-pyrroline-5-carboxylate synthase (P5CS) showed a greater upregulation in the susceptible rice cultivar IR64 (Figure 5).

The transcription response was compared between the tolerant rice cultivar (Apo) and wheat (Weebill). Although ROS-scavenging enzymes FeSOD and CAT showed a marginally greater increase in wheat than in the cultivar Apo, there were no detectable differences in the activity of APX between wheat and the rice cultivar Apo. AKR1 showed significantly higher expression levels in wheat than in cv. Apo. ER stress-responsive genes, CRT1 and BiP1, also showed marginal increases in the transcript level in both tolerant rice (Apo) and wheat (Weebill) cultivars (Figure 5). Fold change in expression of P5CS was significantly higher in wheat compared to that in tolerant rice cv. Apo (Figure 5).

### 3.4. Higher Carbon Gain Helped Wheat to Maintain Higher Growth Rates

**Figure 6:**
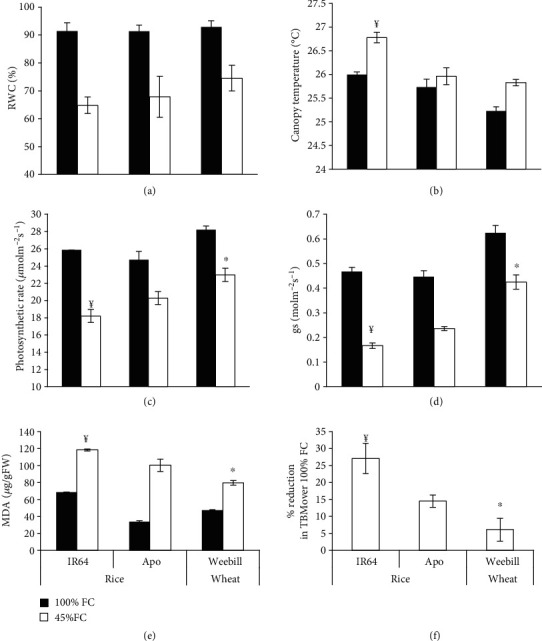
Effect of gradual induction of moisture stress (45% FC) on morphophysiological parameters of IR64 and Apo (rice) and Weebill (wheat) cultivars at the vegetative stage (35 DAS-experiment 2B). (a) Relative water content (RWC). (b) Canopy temperature. (c) Photosynthetic rate. (d) Stomatal conductance (gs). (e) Malondialdehyde (MDA) content was measured during stress. (f) Biomass was measured at 80 DAS and expressed as percent reduction in total biomass (TBM) over 100% FC. ^¥^Significant difference between rice cultivars (IR64 and Apo) in the induction treatment at *p* ≤ 0.05. ^∗^Significant difference between tolerant rice cv. Apo and wheat cv. Weebill in the induction treatment at *p* ≤ 0.05.

At the same soil moisture level of 45% FC, both cereals had lower RWC compared to 100% FC (Figure 6(a)). The automated irrigation system maintained the soil moisture status precisely and at the prescribed level irrespective of differences in water mining (roots) and transpiration. Despite similar RWC, cv. Apo maintained a lower canopy temperature at 45% FC compared to cv. IR64. However, in comparison with tolerant rice cv. Apo, wheat had marginally lower canopy temperature (Figure 6(b)). Significantly greater assimilation rate and stomatal conductance were maintained in wheat at both 100 and 45% FC than in rice cv. Apo (Figures 6(c) and 6(d)). Among rice cultivars, susceptible rice cv. IR64 exhibited significantly lower photosynthetic rate and stomatal conductance at 45% FC. As a stress effect, the percent increase in MDA content was significantly lower in wheat than in the drought-tolerant rice cv. Apo (Figure 6(e)). Of the rice cultivars, MDA content was significantly higher in cv. IR64 than in Apo at 45% FC. Total biomass was measured on 80 DAS; the percent reduction in total biomass was least in the wheat cultivar Weebill, and it was highest in the rice cultivar IR64. Between the rice cultivars, Apo maintained better carbon gain and hence more biomass accumulation even under 45% FC (Figure 6(f)).

## 4. Discussion

Plants have naturally evolved several mechanisms to survive under stressful conditions. A number of mechanisms have been ascribed to these survival strategies, which range from morphological traits associated with maintenance of tissue turgor to physiological mechanisms that help maintain metabolism. Most of these traits are constitutively present and sometimes may also be responsive to stresses [4]. While these constitutive traits have been shown to provide stress adaptation, a set of traits, often referred to as “acquired” tolerance traits, also are known to provide significant levels of tolerance to stresses. These acquired drought tolerance mechanisms or traits are known to be upregulated or expressed only when the plants experience stress, unlike the constitutive traits. In natural conditions, most abiotic stresses like drought and temperature progress gradually and plants would initially experience a mild level of stress and have an opportunity to trigger an acquired response [33]. It is well documented that an array of genes is upregulated when plants experience a progressive stress and mechanisms regulated by these genes subsequently provide tolerance when the stress aggravates and becomes severe [13, 14]. These acquired mechanisms are well documented for temperature tolerance [13, 34]. Studies have shown that seedlings of the induction treatment had a higher recovery than those exposed directly to the severe stress treatment. In an earlier study with diverse rice genotypes, Raju et al. [9] reported that the genotypes that had better acquired tolerance (they referred to this trait as cellular level tolerance) maintained higher spikelet fertility. Thus, it can be visualised that a genotype that has a higher propensity to respond to stress at the young seedling stage would also reflect a higher level of tolerance to stress at later phenological stages as well [9].

Breeding programs predominantly selected genotypes with higher absolute yields under stress with the premise that high yields under stress would result in an automatic selection of acquired tolerance traits. Although this argument is acceptable, there are significant genotypic and species variations in the mechanisms that govern acquired tolerance. However there was not much research on understanding the component mechanisms leading to drought adaptation through acquired tolerance. The major aim of this investigation was, therefore, to examine the variability in acquired tolerance levels between drought-susceptible (IR64) and drought-tolerant (Apo) rice cultivars and a tolerant wheat cultivar (Weebill). Since wheat can sustain better growth and productivity under water-limited conditions compared to rice, we aimed to understand the differences in the acquired tolerance levels between a wheat cultivar (Weebill) and the two rice cultivars.

### 4.1. Upregulation of Protective Mechanisms Helps in Acquiring Better Tolerance in Induction Treatment

Capturing the variability in acquired traits or mechanisms depends on accurate phenotyping for the responses coupled with precise methods to impose induction and severe stress treatments. Many compounds such as methyl viologen [23], DTT [34], NaCl [35], and Rose Bengal [36] have been used to induce stress and capture genetic variability in stress response. Most of these molecules as well as naturally occurring stresses are known to induce an oxidative burst through the generation of reactive oxygen species (ROS). Oxidative stress in turn induces membrane damage and inhibits most metabolic activities, thereby leading to reduced growth rates. Therefore, to maintain the cellular homeostasis, it is essential to either decrease the production of ROS or develop abilities to effectively scavenge and ultimately detoxify the ROS by antioxidants or scavenging enzymes [4, 37]. Further, it is also demonstrated that increases in such scavenging enzymes and antioxidants were high only when plants experienced mild induction treatment compared to plants exposed directly to a severe stress treatment. Hence, rice seedlings directly exposed to the severe stress treatment did not show any recovery growth (Figures 2 and 3). Therefore, it is plausible that variability in production and scavenging mechanisms would determine the susceptibility and tolerance mechanisms of a cultivar. In our study, the susceptible cultivar IR64 showed higher ROS and RCC level damage with lower recovery growth in the induction treatment than the tolerant cv. Apo. Although scavenging mechanisms were comparable between wheat cv. Weebill and tolerant rice cv. Apo, the ROS levels were much lower for wheat, which hence showed better recovery in the induction treatment (Table 1). Recovery hardly occurred after the direct severe stress treatment.

### 4.2. Precise Maintenance of Soil Moisture Is Essential to Capture ADT Traits

Lack of precise stress-imposing methods increased the difficulty in understanding these acquired mechanisms of stress response. The most critical requirement in capturing genetic variability in acquired drought tolerance depends on the ability to maintain the same levels of stress to plants irrespective of differences in evapotranspiration. To capture the induction response, the current study was done using a phenotyping platform with an automated irrigation facility that dispensed precise volumes of water. The volume of water to be dispensed was determined gravimetrically in real time, and the automated solenoid system dispenses water when the soil moisture status decreased by 1% of the set field capacity. Thus, it was possible to precisely maintain the same level of soil moisture in containers irrespective of differences in transpiration and water mining (Figure 1). Accordingly, both cereals showed similar leaf tissue water status at 100% FC and at 45% FC irrespective of their differences in leaf area and root traits (Table 2). This task could only be achieved when the containers were irrigated very frequently based on gravimetric determination of water loss. The automated system was also equipped with a software-controlled stress imposition protocol that permitted the maintenance of a gradual water loss regime until the containers reached the target of 45% FC from the well-watered status of 100% FC. To achieve this, the software commanded the solenoid valves to dispense only a calculated fraction of the transpired water. This setup provided an excellent option to create gradual induction stress treatment before reaching a severe stress level.

Although tissue water status was similar in both cereals, the reduction in total biomass was significantly different. The rice variety IR64 recorded a higher tiller number in 100% FC, and reduction due to stress was significantly higher. Hence, differences in reduction of biomass were mainly due to the variability in acquired drought-tolerant mechanisms (Figure 4).

### 4.3. Differences in ROS Balance and Carbon Assimilation Determine Growth under Stress

Imbalance between ROS production and scavenging leads to the generation of a class of secondary radicals referred to as reactive carbonyl compounds (RCCs)through their reactions with lipids and proteins [38, 39]. These RCCs undergo several modifications with proteins by forming Schiff's bases, Maillard products, and Amadori compounds. ROS-scavenging enzymes like SOD and APX provide the first line of defence against oxidative stress. Similarly, RCC-scavenging enzymes like aldo-keto reductases (AKR) catalyze the reduction of aldehydes and ketones to their respective alcohol using the electrons from NADPH [26, 40]. Besides these ROS- and RCC-detoxifying strategies, plants have also developed mechanisms to overcome oxidative stress damage through protecting proteins through folding and unfolding mechanisms. A stress-responsive gene, BiP1, is involved in protein folding and protection [34]. The other unfolded protein pathway gene, CRT1, is known to bind to Ca^2+^ in ER and acts as a calcium buffer and helps in the folding of newly synthesized proteins [34].

The rice cv. Apo had higher expression of these scavenging enzymes and unfolded protein response than cv. IR64 (Figure 5) and hence showed lower ROS level and better recovery growth. Although wheat is more tolerant than rice, a fold increase in expression of these scavenging enzymes and drought-responsive genes was only marginally higher than that in the drought-tolerant rice cultivar Apo (Figure 5). This could be due to lower levels of ROS production in wheat by reducing excess electron or a better quenching mechanism [41] or by better photochemistry with higher carbon assimilatory capacity. With similar tissue water status, higher photosynthetic rate and higher stomatal conductance might be additional advantages for wheat to maintain higher growth in stress conditions compared with the drought-tolerant rice cv. Apo (Figure 6).

## 5. Conclusion

Expression of acquired tolerance traits is highly dependent on the method of stress imposition. Most reports adopt withholding of water to container-grown plants and record observations when the stress levels reach the target FC. This approach causes a sudden buildup of stress which neither happens in natural field conditions nor gives an opportunity for the plants to stimulate acquired response. Therefore, recovery growth of rice seedlings exposed directly to a severe stress completely retarded compared to induction. Mimicking the stress conditions that occur naturally is, therefore, an important criterion while studying acquired drought responses in plants. In this background, the system used for creating stress is novel and quite precise in creating a gradual drought stress to container-grown plants. This system is also capable of creating a specific and comparable stress regimen independent of differences in transpiration rates between cultivars.

Balancing between ROS production and detoxification is crucial for survival and growth under stress or on its alleviation. Of the two rice cultivars tested, cv. Apo had higher tolerance to stress with higher expression of scavenging enzymes and drought-responsive genes. Wheat seemed to have two strategies: (1) lower production of ROS and RCC through the maintenance of tissue turgor and (2) increasing scavenging activity to detoxify free radicals to maintain redox homeostasis. Although cv. Apo seemed to have good scavenging mechanisms, they were not sufficient to detoxify the total ROS produced. Hence, our findings reveal that understanding and further dissecting the acquired drought-tolerant mechanism though a precise stress imposition protocol and between-species comparison will help in crop improvement. Improving these acquired mechanisms along with adaptive traits through precise phenotyping for these traits would be very crucial while developing rice varieties suitable for cultivation under the semi-irrigated aerobic conditions.

## Figures and Tables

**Table 1:** Effects of methyl viologen on ROS and RCC production in seedlings of two rice cultivars and one wheat cultivar (Experiment 1). Accumulation of superoxide (O_2_^−^), hydroxyl radical (^∙^OH), malondialdehyde (MDA), and methylglyoxal (MG) contents and extent of membrane damage measured by the Evans blue technique in control, induction, and severe (10 *μ*M) treatments of methyl viologen. ^†^Significant difference between induction and severe stress treatments within cultivars at *p* ≤ 0.05. ^¥^Significant difference between rice cultivars (IR64 and Apo) in the induction treatment at *p* ≤ 0.05. ^∗^Significant difference between tolerant rice cv. Apo and wheat cv. Weebill in the induction treatment at *p* ≤ 0.05.

Parameters	Treatment	Rice		Wheat
		IR64	Apo	Weebill
O_2_^−^ (Absor × 1000)	Control	127 ± 9.6	122 ± 6.0	104 ± 7.2
	Induction	955 ± 37.6^¥^	836 ± 18.4	601 ± 19.1^∗^
	10 *μ*M MV (severe)	1668 ± 45.5^†^	1268 ± 39.4^†^	916 ± 23.4^†^

^∙^OH (Absor × 1000)	Control	26 ± 2.1	29 ± 2.1	31 ± 5.5
	Induction	141 ± 2.8^¥^	102 ± 2.0	101 ± 5.0
	10 *μ*M MV (severe)	275 ± 7.1^†^	218 ± 1.4^†^	173 ± 4.6^†^

MDA (*μ*g/gFW)	Control	12.6 ± 4.5	12.8 ± 1.5	18.6 ± 1.5
	Induction	59.1 ± 0.9	55.2 ± 0.3	45.0 ± 1.1
	10 *μ*M MV (severe)	62.3 ± 0.9^†^	58.8 ± 1.0^†^	53.0 ± 0.4^†^

MG (*μ*g/gFW)	Control	13.3 ± 1.5	18.6 ± 3.2	15.6 ± 3.5
	Induction	52.0 ± 2.6^¥^	45.3 ± 1.5	35.6 ± 3.0^∗^
	10 *μ*M MV (severe)	78.0 ± 1.0^†^	65.3 ± 4.0^†^	56.3 ± 4.1^†^

Membrane damage (ngEvansblue/gFW)	Control	0.7 ± 0.01	0.6 ± 0.15	0.6 ± 0.01
	Induction	3.1 ± 0.32^¥^	2.0 ± 0.08	1.8 ± 0.08
	10 *μ*M MV (severe)	5.1 ± 0.34^†^	3.9 ± 0.25^†^	3.7 ± 0.05^†^

**Table 2:** Effects of gradual imposition of drought stress on physiological parameters of two rice cultivars (IR64 and Apo) and one wheat cultivar (Weebill) (Experiment 2A). Gradual drought stress was imposed at 15 DAS using an automated irrigation and plant phenotyping platform. Physiological parameters like relative water content (RWC), total chlorophyll, malondialdehyde (MDA), and total proline were measured at 100% FC and 45% FC conditions. ^¥^Significant difference between rice cultivars (IR64 and Apo) in the induction treatment at *p* ≤ 0.05. ^∗^Significant difference between tolerant rice cv. Apo and wheat cv. Weebill in the induction treatment at *p* ≤ 0.05.

Parameters	Treatment	Rice		Wheat
		IR64	Apo	Weebill
RWC (%)	100% FC	91.6 ± 2.4	93.4 ± 2.9	88.0 ± 0.9
	45% FC	60.4 ± 5.4	62.5 ± 3.5	62.6 ± 1.2
Total chlorophyll (mg/gFW)	100% FC	0.8 ± 0.02	0.7 ± 0.06	1.3 ± 0.07
	45% FC	0.6 ± 0.01	0.6 ± 0.06	1.0 ± 0.12
MDA (*μ*g/FW)	100% FC	14.4 ± 1.8	63.1 ± 6.5	29.3 ± 3.4
	45% FC	315.4 ± 3.7^¥^	265.7 ± 6.9	247.0 ± 19.7^∗^
Total proline (*μ*g/gFW)	100% FC	75.1 ± 24.1	76.2 ± 6.03	126 ± 5.3
	45% FC	396.4 ± 14.8^¥^	259.5 ± 24.4	408.4 ± 33.6

## Supplementary Materials

Supplementary Materials Fig. S1: standardization of severe dose of MV using the rice cultivar IR64. Seedlings were directly transferred to 2, 4, 6, and 8 *μ*M, and observations were recorded 48 hours after stress imposition. Table S1: weather parameters during experiment 2A. Table S2: weather parameters during experiment 2B. Table S3: percent reduction in growth of rice and wheat seedlings due to MV-induced stress (a) at the end of the stress period and (b) after recovery (Experiment 1).
