# Supplementary material for: Acquired Traits Contribute More to Drought Tolerance in Wheat Than in Rice
Source: Plant Phenomics. 2020 Jun 12;2020:5905371. doi: 10.34133/2020/5905371 (PMC7706322; doi:10.34133/2020/5905371)

Table S3: Percent reduction in growth of rice and wheat seedlings due to MV induced stress at (a) end of stress period and (b) after recovery.


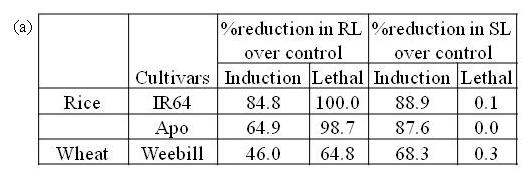


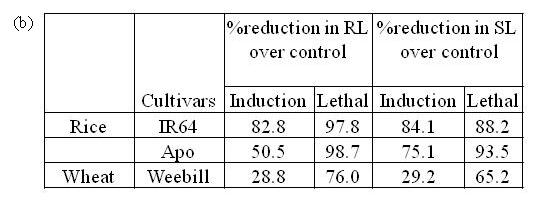

Supplement: Supplementary Materials — Fig. S1: standardization of severe dose of MV using the rice cultivar IR64. Seedlings were directly transferred to 2, 4, 6, and 8 μM, and observations were recorded 48 hours after stress imposition. Table S1: weather parameters during experiment 2A. Table S2: weather parameters during experiment 2B. Table S3: percent reduction in growth of rice and wheat seedlings due to MV-induced stress (a) at the end of the stress period and (b) after recovery (Experiment 1). [file 5905371.f1.zip › Suppl Table S3.docx]
